# Selection against BALB/c strain cells in mouse chimaeras

**DOI:** 10.1242/bio.030189

**Published:** 2018-01-15

**Authors:** Pin-Chi Tang, Gillian E. MacKay, Jean H. Flockhart, Margaret A. Keighren, Anna Kopakaki, John D. West

**Affiliations:** 1 Genes and Development Group, Centre for Integrative Physiology, Clinical Sciences, University of Edinburgh Medical School, Hugh Robson Building, George Square, Edinburgh, EH8 9XD, UK; 2 Obstetrics and Gynaecology Section, Clinical Sciences, University of Edinburgh Medical School, The Queen's Medical Research Institute, 47 Little France Crescent, Edinburgh, EH16 4TJ, UK

**Keywords:** BALB/c mouse strain, Mouse embryo, Cell selection, Chimaera, Chimera

## Abstract

It has been shown previously that BALB/c strain embryos tend to contribute poorly to mouse aggregation chimaeras. In the present study we showed that BALB/c cells were not preferentially allocated to any extraembryonic lineages of mouse aggregation chimaeras, but their contribution decreased during the early postimplantation period and they were significantly depleted by E8.5. The development of BALB/c strain preimplantation embryos lagged behind embryos from some other strains and the contribution that BALB/c and other embryos made to chimaeras correlated with their developmental stage at E2.5. This relationship suggests that the poor contribution of BALB/c embryos to aggregation chimaeras is at least partly a consequence of generalised selection related to slow or delayed preimplantation development. The suitability of BALB/c embryos for maximising the ES cell contribution to mouse ES cell chimaeras is also discussed.

## Abstract (precis)

**Summary:** BALB/c strain embryos contributed poorly to mouse aggregation chimaeras by E8.5. Selection appears linked to slow BALB/c development and might also explain the good ES cell contribution in BALB/c↔ES-cell chimaeras.

## INTRODUCTION

Mouse aggregation chimaeras provide powerful research tools for a wide range of investigations (Eckardt et al., 2011), but the relative contribution of the two aggregated embryos varies widely among individual adult chimaeras and is difficult to control. This variation is useful for the analysis of the effects of genetic mutants because phenotypes can be correlated with the composition of chimaeric tissues. However, for spatial analysis of clonal growth and cell mixing, it would be more advantageous to control the variation and produce mainly chimaeras with only a small proportion of cells that carry a lineage marker. It has long been known that the composition of chimaeras is affected by the strain combination, but the basis for this bias remains poorly understood. One early study showed that C57BL/10 strain melanocytes contributed more than BALB/c melanocytes to the coats of most chimaeras in a series of BALB/c↔C57BL/10 aggregation chimaeras (Mullen and Whitten, 1971). BALB/c cells also contributed poorly to several series of embryonic day (E) 12.5 chimaeric conceptuses analysed by glucose phosphate isomerase (GPI) electrophoresis (West and Flockhart, 1994; Tang and West, 2001; MacKay et al., 2005). Relevant results from four of our published E12.5 chimaera studies are summarised as experiments 1-4 in Fig. S1 and Tables 1 and 2, and data are archived (Flockhart et al., 2017). There are two series of E12.5 chimaeras in each experiment; one is a balanced strain combination (series B) and the other is a more unbalanced strain combination (series U).

These experiments showed that BALB/c cells tended to contribute poorly to the foetus, amnion and yolk sac mesoderm (which are all derived from the epiblast), and also relatively poorly to the yolk sac endoderm and placenta (Fig. S1A-C; Table 2) (West and Flockhart, 1994; Tang and West, 2001; MacKay et al., 2005). The yolk sac endoderm is derived from the primitive endoderm (PrE). Placental GPI would have been almost entirely from the polar trophectoderm (pTE) lineage because other lineages only contribute about 4% to the placenta (Rossant and Croy, 1985), and maternal GPI1C was excluded by electrophoresis. If the poor contribution of BALB/c cells to the epiblast lineage was caused entirely by preferential allocation of BALB/c cells to other lineages, BALB/c cells should be over-represented in those lineages. BALB/c cells were not over-represented in the yolk sac endoderm or placenta in E12.5 chimaeras, and there was usually a clear trend for them to be under-represented in these lineages even if this was less often significant than in the epiblast tissues (Fig. S1A-C; Table 2) (West and Flockhart, 1994; Tang and West, 2001; MacKay et al., 2005). The poor contribution of BALB/c cells to the epiblast lineage cannot, therefore, be explained by preferential allocation of BALB/c cells to the PrE or pTE lineages. However, the mural trophectoderm (mTE) lineage could not be analysed at this stage because mTE cells stop dividing early in development, and so contribute few cells to the mid-gestation mouse conceptus (Gardner and Papaioannou, 1975).

The E12.5 chimaera results suggest that BALB/c cells could be either preferentially allocated to the mTE lineage or at some general selective disadvantage. Moreover, analysis of chimaeras that were produced by aggregating pairs of half embryos showed that the low contribution of BALB/c cells did not depend on events that occurred as part of the size regulation process during chimaera development (Tang and West, 2001). A possible basis for generalised selection against BALB/c cells in aggregation chimaeras is suggested by studies reporting that the preimplantation development of BALB/c embryos lagged behind embryos of some other strains (Gates et al., 1961; Whitten and Dagg, 1961; Goldbard and Warner, 1982), and were still delayed relative to 129/Rr embryos at E9.5 (Dagg, 1960).

Another experiment showed that backcross embryos with BALB/c mothers contributed more poorly to chimaeras than reciprocal backcross embryos (West et al., 1995). Although this difference failed to reach significance in the analysis shown as E12.5 experiment 2 in Fig. S1D, the compositions of the two series of chimaeras did differ significantly when tested against the expectations for a balanced strain combination (West et al., 1995). This difference suggested the possibility of a BALB/c maternal effect on chimaera composition.

The aim of the present study was to determine why BALB/c embryos make a poor contribution to aggregation chimaeras. To test the hypotheses that BALB/c cells are preferentially allocated to the mTE lineage, we investigated whether BALB/c cells were better represented in the mTE than other regions of chimaeric blastocysts. To test the hypotheses that BALB/c cells are at a general selective disadvantage in chimaeras, we identified when depletion of BALB/c cells in chimaeras was first detected and tested whether the contribution that BALB/c and other embryos made to E12.5 chimaeras was correlated with their mean stage of development at E2.5 and E3.5.

## RESULTS

### BALB/c cells are not preferentially allocated to the mural trophectoderm lineage

**Fig. 1.**
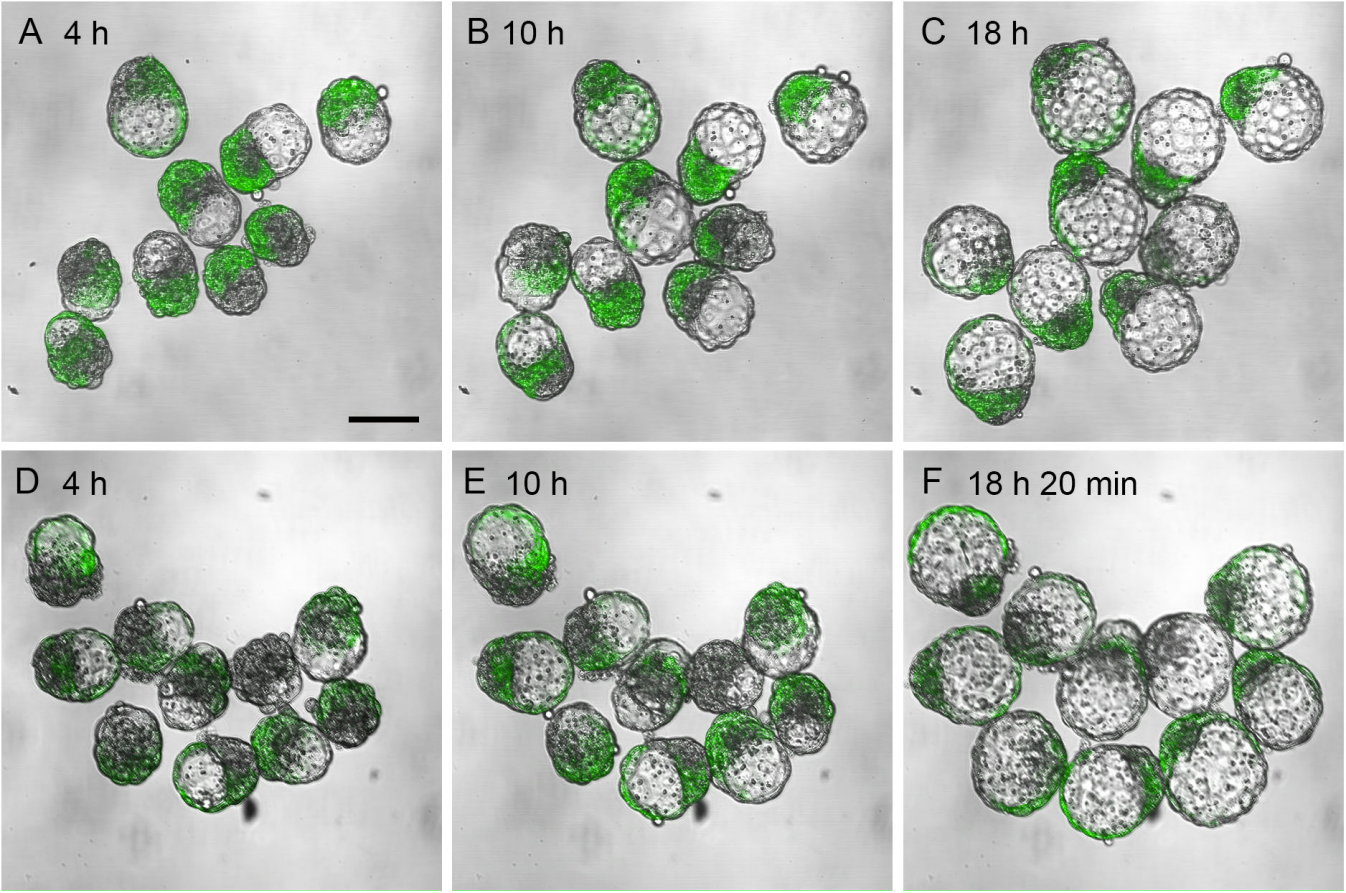
**Time-lapse confocal microscope images of chimaeric blastocysts marked with tauGFP.** Embryos were aggregated at E2.5 and incubated for 24 h in 5% CO_2_ in air in a closed incubator and then transferred to an environmental chamber on the stage of a confocal microscope. (A-C) Merged fluorescence and transmitted light time-lapse images of chimaeric blastocysts from the balanced strain combination AAF2↔(BF1×TP6.3) (blastocyst chimaera series 4B) after 4 h (A), 10 h (B) and 18 h (C) in the environmental chamber. (D-F) Time-lapse images of chimaeric blastocysts from the unbalanced strain combination BALB/c↔(BF1×TP6.3) (blastocyst chimaera series 4U) after 4 h (D), 10 h (E) and 18 h 20 min (F) in the environmental chamber. Scale bar: 100 µm.

**Fig. 2.**
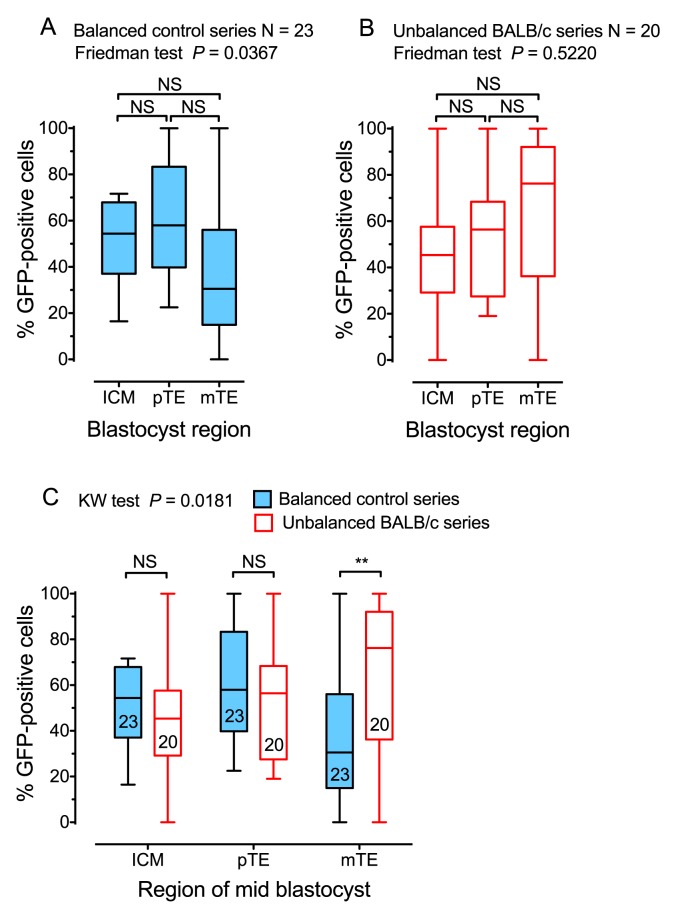
**Contribution of GFP-positive cells to two series of chimaeric blastocysts.** The composition of chimaeric blastocysts from the balanced strain combination AAF2↔(BF1×TP6.3) (blastocyst series 4B) and unbalanced strain combination BALB/c↔(BF1×TP6.3) (blastocyst series 4U) were compared. The (BF1×TP6.3) cells were positive for the TP6.3 tauGFP marker. Results shown here were obtained by imaging embryos at the mid-blastocyst stage and data were pooled from two experiments. (A,B) The composition of different blastocyst regions, within each series, were compared by the Friedman test (*P*-values are shown) and Dunn's multiple comparison test (none was significant). (C) The composition of balanced versus unbalanced series for each region were compared by the Kruskal–Wallis (KW) test (*P*-values are shown) and Dunn's multiple comparison test (significant results are shown by asterisks). ICM, inner cell mass; mTE, mural trophectoderm; pTE, polar trophectoderm. **P<0.01; NS, not significant. The number of blastocysts is shown within the box and whisker plots in C. Box and whisker plots show the median (horizontal line within the box), upper and lower quartiles (top and bottom of boxes) and the minimum and maximum of all the data (ends of whiskers).

To investigate whether BALB/c cells were preferentially allocated to the mTE in chimaeras, we analysed the composition of the inner cell mass (ICM), pTE (overlying the ICM) and mTE (surrounding the blastocyst cavity) in chimaeric blastocysts, carrying the *TgTP6.3* tauGFP marker transgene (Pratt et al., 2000; MacKay et al., 2005). These were made with the same strain combinations as E12.5 unbalanced (U) series 4U and balanced (B) series 4B (Tables 1 and 2). The tauGFP marker labelled the partner (BF1×TP6.3) embryos rather than the BALB/c embryos in series 4U chimaeras, or the AAF2 embryos in series 4B chimaeras. To allow all the chimaeric blastocysts to be analysed at equivalent stages, they were cultured in an environmental chamber on the stage of a confocal microscope (Sharp et al., 2017) and imaged at intervals (Fig. 1). The percentage of (BF1×TP6.3) tauGFP-positive cells was estimated separately for the ICM, pTE and mTE at each of three blastocyst stages, but there were no significant differences among developmental stages by Friedman tests (data not shown). Fig. 2A-C shows results for mid-blastocyst stage chimaeras that were pooled from two experiments.

**Table 1.**
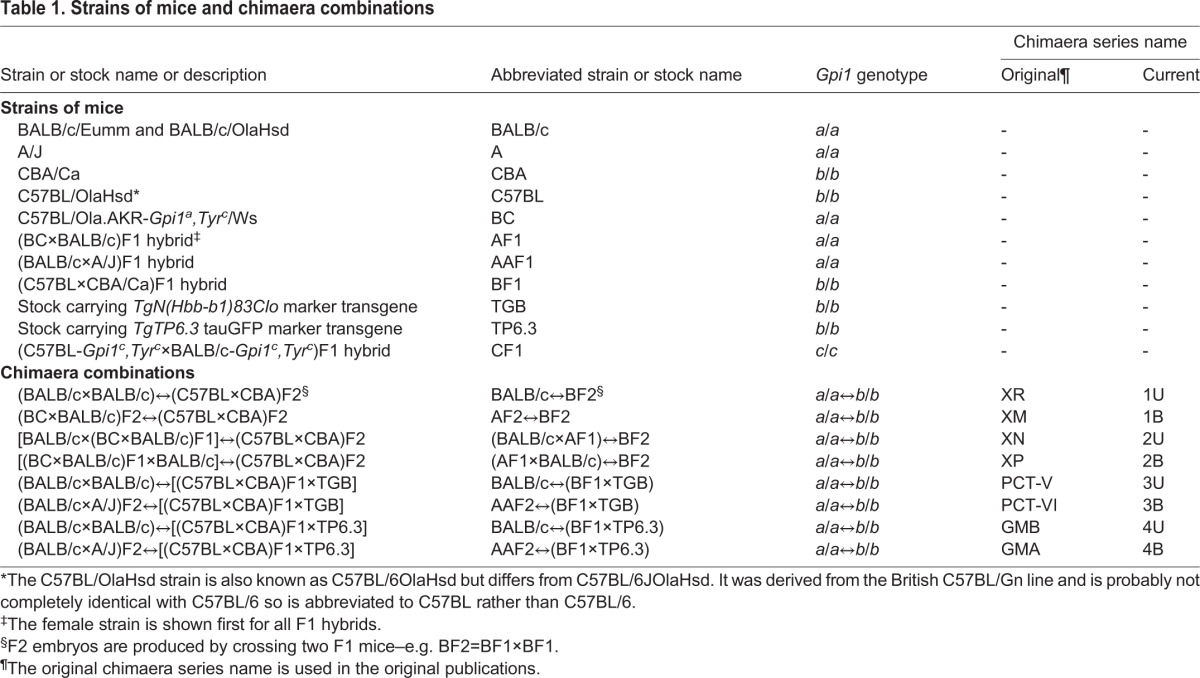
**Strains of mice and chimaera combinations**

**Table 2.**
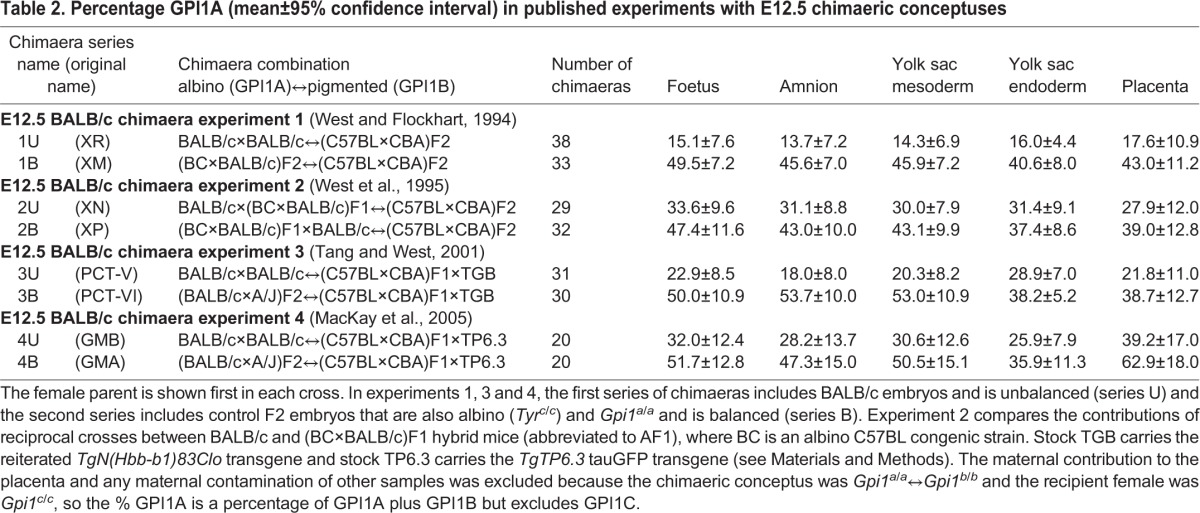
**Percentage GPI1A (mean±95% confidence interval) in published experiments with E12.5 chimaeric conceptuses**

Contributions of tauGFP-positive (BF1×TP6.3) cells did not differ significantly among mTE, pTE and ICM regions of the chimaeric blastocysts in the balanced AAF2↔(BF1×TP6.3) strain combination 4B (Fig. 2A) or the unbalanced BALB/c↔(BF1×TP6.3) strain combination 4U (Fig. 2B). Comparisons between the two series of chimaeric blastocysts showed that there was a higher proportion of tauGFP-positive (BF1×TP6.3) cells in the mTE of the BALB/c↔(BF1×TP6.3) chimaeras from series 4U than in the AAF2↔(BF1×TP6.3) chimaeras from the more balanced series 4B (Fig. 2C). These results suggest that BALB/c cells tend to be depleted from the mTE and clearly provide no evidence that they are preferentially allocated to the mTE. The contribution of tauGFP-positive (BF1×TP6.3) cells to the ICM was not significantly higher in chimaeric blastocysts in the unbalanced series 4U than in those from the balanced series 4B (Fig. 2C). This suggested the possibility that the BALB/c cells are mainly depleted from the ICM lineage of chimaeras after the blastocyst stage.

### Selection against BALB/c cells

**Fig. 3.**
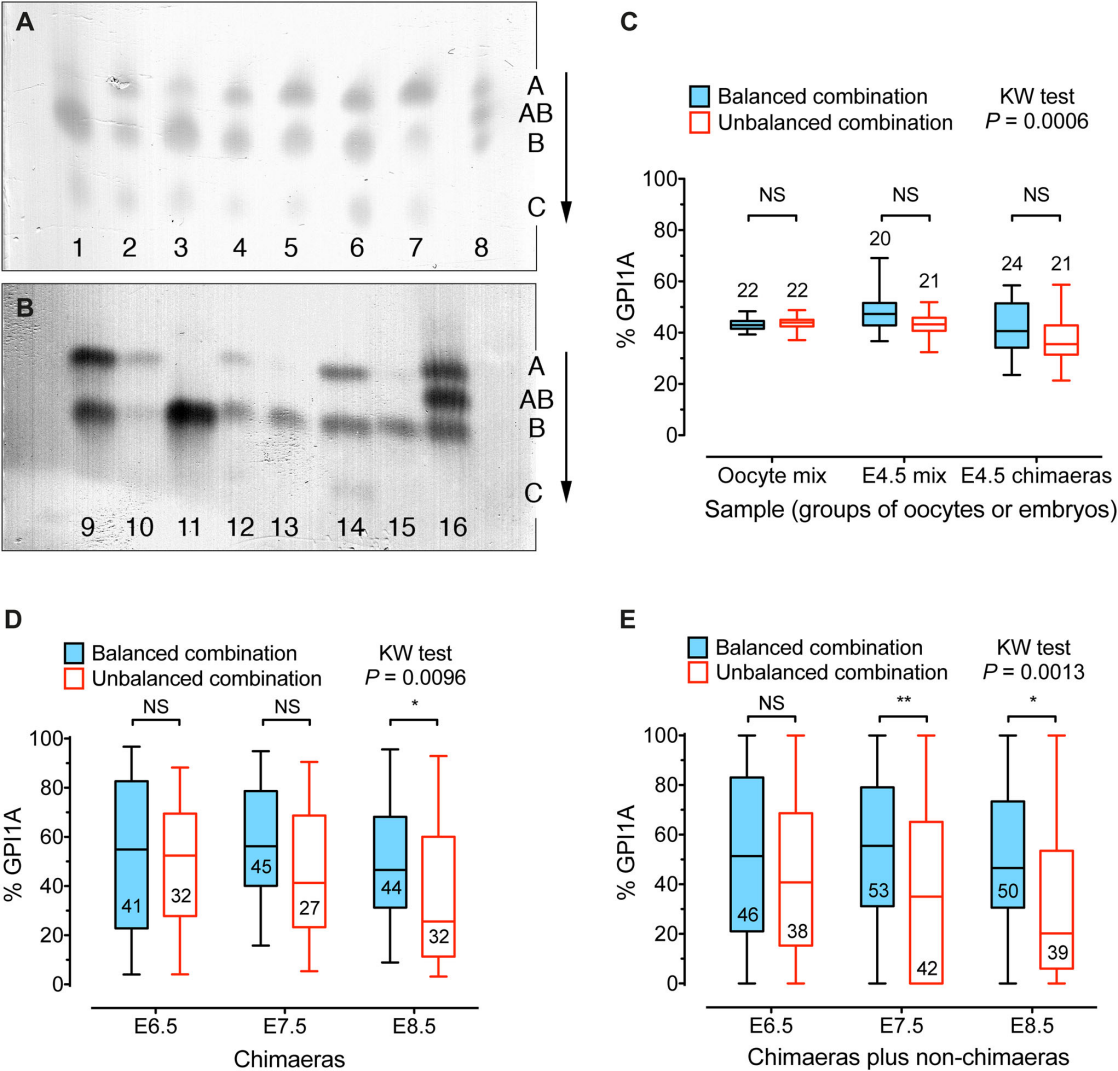
**Contribution of GPI1A cells to chimaeras aged E4.5 to E8.5.** (A,B) Electrophoresis of GPI in E7.5 (A) and E8.5 (B) *Gpi1^a/a^*↔*Gpi1^b/b^* chimaeras from the balanced strain combination. Lanes 1-7 are E7.5 chimaera samples and lanes 9-15 are E8.5 chimaera samples. Lanes 8 and 16 are blood samples from *Gpi1^a/b^* heterozygotes used as a reference. The GPI1C band is maternal and was excluded from the estimation of the percentage GPI1A. Migration was in the direction of the arrow. Abbreviations of GPI allozyme bands: A, GPI1AA homodimer; AB, GPI1AB heterodimer; B, GPI1BB; C, GPI1CC. (C) Comparison of the percentage GPI1A in mixtures of six oocytes (‘oocyte mix’), mixtures of six E4.5 blastocysts (‘E4.5 mix’) and groups of three E4.5 chimaeras. The oocyte mixtures, representing balanced series 3B, comprised three AAF1 plus three BF1 oocytes and those, representing the unbalanced series 3U, comprised three BALB/c plus three BF1 oocytes. The E4.5 blastocyst mixtures, representing balanced series 3B, comprised three AAF2 plus three (BF1×TGB) blastocysts and those, representing the unbalanced series 3U, comprised three BALB/c plus three (BF1×TGB) blastocysts. The E4.5 chimaeras were from series 3B and 3U and each sample comprised three E4.5 chimaeric blastocysts. (D,E) Comparison of the percentage GPI1A in individual E6.5, E7.5 and E8.5 chimaeras from series 3B and 3U. Non-chimaeras were excluded from D but included in E. The percentage GPI1A was compared by the Kruskal–Wallis (KW) test (*P*-values are shown) and Dunn's multiple comparison test for pairwise comparisons between series 3B and 3U. *P<0.05; **P<0.01; NS, not significant. The number of samples is shown either above or within the box and whiskers plots. Box and whisker plots show the median (horizontal line within the box), upper and lower quartiles (top and bottom of boxes) and the minimum and maximum of all the data (ends of whiskers).

We next investigated when BALB/c cells became detectably under-represented in chimaeras. We compared the overall composition of two series of *Gpi1^a/a^*↔*Gpi1^b/b^* chimaeric conceptuses between E4.5 and E8.5 by GPI1 electrophoresis (Fig. 3A,B) using the same strain combinations as those shown as E12.5 series 3B and 3U in Tables 1 and 2. The *Gpi1^a/a^* genotype identified the BALB/c cells in the unbalanced chimaera series 3U and the AAF2 cells in the balanced series 3B. Oocyte GPI activity varies among mouse strains (Peterson and Wong, 1978; West and Fisher, 1984), so we first confirmed that the relative GPI activity did not differ significantly between oocytes from strains that were used as females to produce embryos for the balanced and unbalanced series of chimaeras (series 3B and 3U, respectively). For this, we compared the % GPI1A in mixtures of three AAF1 (*Gpi1^a/a^*) plus three BF1 (*Gpi1^b/b^*) oocytes with that in mixtures of three BALB/c (*Gpi1^a/a^*) plus three BF1 oocytes (‘oocyte mix’ in Fig. 3C). We also showed that there was no significant difference in the percentage of GPI1A between mixtures of three AAF2 plus three (BF1×TGB) embryos and mixtures of three BALB/c plus three (BF1×TGB) embryos. The embryos were cultured from E2.5 to E4.5 then mixed, to represent the strain combinations present in the aggregated embryos from balanced chimaera series 3B and unbalanced series 3U, respectively (‘E4.5 mix’ in Fig. 3C).

Direct comparisons of groups of three E4.5 chimaeric blastocysts, showed that the percentage of GPI1A was not significantly less in the unbalanced BALB/c↔(BF1×TGB) series 3U than in the balanced AAF2↔(BF1×TGB) series 3B at this stage (E4.5 chimaeras in Fig. 3C). As expected, the percentage of GPI1A in individual E6.5, E7.5 and E8.5 chimaeras was more variable than in the groups of three E4.5 chimaeras (Fig. 3), and the extent of variation resembled that shown among individual E12.5 chimaeras (Fig. S1). There were trends for more E6.5-E8.5 conceptuses in the unbalanced series 3U to be non-chimaeric and for more of these to be 0% GPI1A, rather than 100% GPI1A, than in the balanced series 3B (Table S1). The mean percentage of GPI1A was significantly less in the unbalanced series of BALB/c↔(BF1×TGB) chimaeras than in the balanced series of AAF2↔(BF1×TGB) chimaeras by E8.5, if the non-chimaeras were excluded, or by E7.5, if the non-chimaeras were included (Fig. 3D,E). These results show that BALB/c cells were not significantly depleted in BALB/c↔(BF1×TGB) chimaeras at E4.5 but were depleted by E8.5, implying that they were at a selective disadvantage between E4.5 and E8.5.

### Delayed or slow development of BALB/c preimplantation embryos

We compared the development of embryos from the strains used to produce the E12.5 chimaeras, shown in chimaera experiments 1-3 in Table 2, in three embryo development experiments. Each embryo development experiment compared the development of embryos of three strains (two *Gpi1^a/a^* and one *Gpi1^b/b^* strain) used to produce the two chimaera series in the corresponding chimaera experiment. For example, embryo development experiment 1 compared the embryonic development of the three strains used to produce E12.5 chimaeras series 1U and 1B in chimaera experiment 1. Embryos were collected at E2.5 and cultured for 24 h, equivalent to the culture period used to make chimaeras. The overall developmental status of each embryo strain was summarised as the mean developmental score at E2.5 and E3.5, as explained in the Materials and Methods, and the percentage of blastocysts at E3.5. The developmental scores for the *Gpi1^a/a^* and *Gpi1^b/b^* strains used in a series of chimaeras, were used to calculate a relative developmental index for the *Gpi1^a/a^* strain in that chimaera series. The relative developmental index was calculated as the developmental score of the *Gpi1^a/a^* strain, expressed as a percentage of the sum of the scores for the *Gpi1^a/a^* and *Gpi1^b/b^* strains that were combined in the chimaeras (see Materials and Methods).

**Fig. 4.**
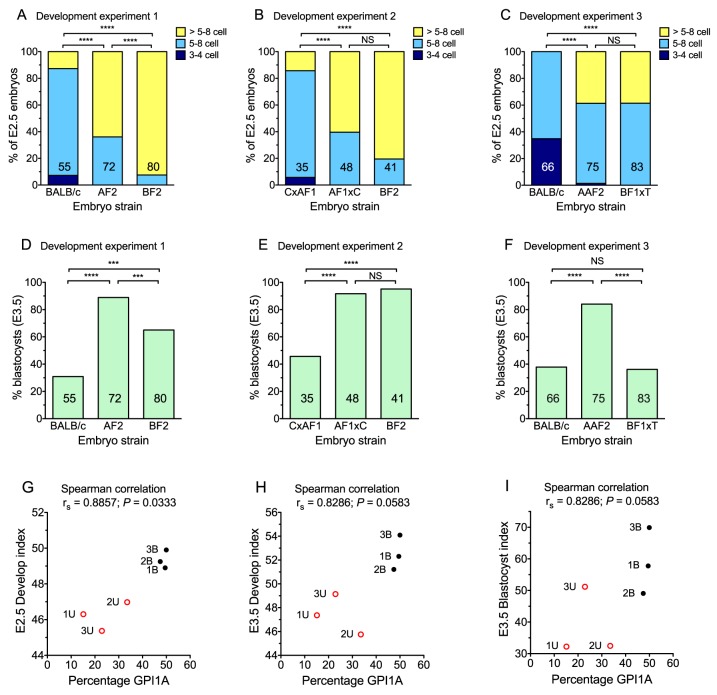
**Relationship between embryo development and percentage GPI1A contribution to E12.5 chimaeric foetuses.** (A-C) Percentage of embryos at the 3-4 cell, 5-8 cell and >5-8 cell stage (either compacting 5-8 cell or >8 cells) that were collected at E2.5 in development experiments 1-3 respectively. The numbers of embryos are shown within the bars and frequencies of embryos at the three different stages were compared between pairs of strains by the 3×2 Fisher's exact test. (D-F) Percentage of embryos that had reached the blastocyst stage after being collected at E2.5 and cultured for 24 h to E3.5, in development experiments 1-3 respectively. The numbers of embryos are shown in the bars and blastocyst frequencies were compared between pairs of strains by the 2×2 Fisher's exact test. For A-F, *P*-values are indicated by asterisks: ***P<0.001; ****P<0.0001; NS, not significant. (G-I) Correlations between the percentage GPI1A in the foetus of E12.5 chimaeras, shown in Table 2, and the E2.5 relative developmental index for the *Gpi1^a/a^* strain (G), the E3.5 relative developmental index for the *Gpi1^a/a^* strain (H) and the E3.5 relative blastocyst index for the *Gpi1^a/a^* strain (I) for the same strain combinations (shown as 1B, 1U, 2B, 2U, 3B and 3U). Spearman correlation coefficients (r_s_) and *P*-values are shown above the graphs. C×AF1, (BALB/c female×AF1 male); AF1×C, (AF1 female×BALB/c male); BF1xT, (BF1 female×TGB male).

When embryos were collected at E2.5, in embryo development experiments 1-3, BALB/c embryos and (BALB/c female×AF1 male) embryos were lagging behind the others (Fig. 4A-C; Table S2) and, for experiments 1 and 2, they remained lagging behind after 24 h in culture (Fig. 4D-F; Table S3). The E2.5 BALB/c embryos in embryo development experiment 3 also lagged behind those in experiment 1, because embryos were collected about 2 h earlier (see Materials and Methods). Fig. 4G shows that the mean percentage of GPI1A for the foetuses of the six series of E12.5 chimaeras, named 1U, 1B, 2U, 2B, 3U, and 3B in Table 2, was significantly positively correlated with the corresponding E2.5 relative developmental index. The mean percentages of GPI1A were also strongly positively correlated (correlation coefficients >0.7) with the E3.5 relative developmental index and the E3.5 relative blastocyst index (based on the percentage of blastocysts at E3.5), but the correlations were not statistically significant (Fig. 4H,I).

Overall, these results imply that the contribution that an embryo makes to the foetus of an E12.5 aggregation chimaera correlates with its stage of development at E2.5. Furthermore, the differences between embryos from the reciprocal crosses between BALB/c and AF1 mice (Fig. 4B,E; Tables S2 and S3) are consistent with a maternal effect contributing both to the slower or delayed development of BALB/c embryos and their poorer contribution to chimaeras.

Finally, to quantify the developmental lag of BALB/c embryos in terms of cell doublings, we compared cell numbers in 139 BALB/c and 127 (BF1×TGB) embryos at E2.5. As expected, the BALB/c embryos had significantly fewer cells than the (BF1×TGB) embryos. Median cell numbers were 6 and 9, respectively, and mean±95% confidence intervals were 6.47±0.34 cells for BALB/c and 9.87±0.47 cells for (BF1×TGB) embryos (*P*<0.0001 by Mann-Witney *U*-test). The mean numbers of cell doublings completed were estimated as 2.69 for BALB/c and 3.30 for (BF1×TGB) embryos, from the log_2_ of the mean cell number, suggesting that BALB/c embryos lagged behind by about 0.61 cell doublings.

## DISCUSSION

Results of our chimaera experiments revealed no evidence that BALB/c cells were preferentially allocated to the mTE but indicated they were at a selective disadvantage between E4.5 and E8.5. We confirmed previous reports that preimplantation BALB/c embryos lagged behind embryos of some other strains and showed that the relative stage of development attained by E2.5 embryos of different strains correlated with the contribution they made to chimaeras. This suggests that the poor contribution of BALB/c embryos to aggregation chimaeras is at least partly because they lag behind their partner embryos, due to delayed or slow development.

In principle, heterosis of non-inbred partner embryos might also have contributed to the poor contribution of BALB/c cells in BALB/c↔BF2, BALB/c↔(BF1×TGB) and BALB/c↔(BF1×TP6.3) chimaeras, if hybrid cells outgrow BALB/c cells. However, this does not explain the differences in compositions of (BALB/c×AF1)↔BF2 and (AF1×BALB/c)↔BF2 chimaeras (West et al., 1995), or the poor contribution of BALB/c cells to adult BALB/c↔C57BL/10 chimaeras (Mullen and Whitten, 1971). Similarly, it does not explain the poor contribution of inbred C3H/HeN strain cells to C3H/HeN↔C57BL/6N chimaeras, which was also not detected until E7.5 (Dvorak et al., 1995).

Like BALB/c embryos, C3H strain embryos often contribute poorly to chimaeras and lag behind embryos of many other strains. This lag may be caused by a combination of delayed fertilisation (McLaren and Bowman, 1973; Nicol and McLaren, 1974; Niwa et al., 1980) and slow development. C3H/He embryos divide relatively slowly because they carry the slow allele of the *Ped* (preimplantation embryo development) gene complex (Verbanac and Warner, 1981; Goldbard et al., 1982; Warner et al., 1998). However, unlike C3H mice, BALB/c mice carry the *Ped-fast* allele, so *Ped* cannot cause the developmental lag of BALB/c preimplantation embryos (Goldbard and Warner, 1982).

As far as we know, the reason why BALB/c preimplantation embryos lag behind those of many other strains is not understood. Our results, showing that (BALB/c female×AF1 male) embryos lag behind reciprocal (AF1 female×BALB/c male) embryos by E2.5, is consistent with their poorer contribution to E12.5 chimaeras (West et al., 1995) and suggests that a maternal effect is involved. However, Whitten and Dagg (1961) showed that BALB/c embryos underwent their first cleavage earlier but subsequently lagged behind 129/Rr strain embryos, so delayed fertilisation seems less likely than slow embryo development that is not caused by the *Ped* genotype.

Goldbard and Warner (1982) estimated mean cell numbers for BALB/c and C57BL/10 embryos as 4.5 and 7.6 cells respectively, at 65 h post hCG, and as 22.0 and 33.1 cells, at 89 h. This suggests that BALB/c embryos lagged by approximately 0.76 cell doublings at 65 h [log_2_(7.6/4.5)] and 0.59 doublings at 89 h [log_2_(33.1/22.0)]. This is comparable to our estimate that BALB/c embryos lagged behind (BF1×TGB) embryos by approximately 0.61 cell doublings at E2.5. Overall these data suggest that BALB/c embryos lagged behind C57BL/10 and (BF1×TGB) embryos by 0.59 to 0.76 cell doublings at these stages. If the cleavage cell cycle time is about 11-12 h, this is equivalent to a lag time of approximately 6-9 h, but this will be imprecise because cell divisions retain some synchrony.

Some evidence suggests that delayed or slow preimplantation development of BALB/c embryos might be predicted to cause a poorer contribution of BALB/c cells to the ICM than the trophectoderm (TE) in aggregation chimaeras. Studies of intact preimplantation mouse embryos (Barlow et al., 1972), or embryos that were dissociated for labelling, then reassembled (Kelly et al., 1978), implied that early dividing cells tended to contribute preferentially to the ICM lineage. After correcting for the effects of the ^3^H-thymidine label on cell numbers, results for giant chimaeric blastocysts, produced by aggregating four embryos, indicated that the more advanced embryo(s) contributed more to the ICM then expected by chance in some, but not all, chimaera combinations (Spindle, 1982). In addition, analysis of other chimaeras, made by aggregating fractions of embryos, supported the conclusion that early dividing cells or cells from older embryos contribute preferentially to the ICM lineage (Surani and Barton, 1984; Garbutt et al., 1987). However, a more recent study with intact embryos found no convincing evidence that early dividing cells tended to contribute preferentially to the ICM lineage (Bischoff et al., 2008).

Furthermore, comparisons of the compositions of E12.5 chimaeras, made with a balanced *Gpi1^a/a^*↔*Gpi1^b/b^* strain combination, showed that younger 4-cell embryos and half embryos at the 8-cell stage both contributed less well than whole 8-cell stage embryos to the foetus, amnion, yolk sac mesoderm, yolk sac endoderm and placenta (Tang and West, 2000). This is consistent with our conclusion that BALB/c embryos contribute relatively poorly to the entire mid-gestation, chimaeric conceptus because they lag behind their partner embryo.

Despite the evidence that BALB/c embryos often lag behind their partner embryos before they are aggregated to produce chimaeras, we did not detect any significant under-representation of BALB/c cells in chimaeras until E8.5. One possibility is that small differences in cell cycle times that persisted after implantation could cause a continuous but small selection pressure against BALB/c cells, such that the cumulative effects take time to reach a detectable level. Another possibility is that a difference in preimplantation cell numbers, between the BALB/c embryo and its partner, is amplified after implantation when the epiblast cell cycle shortens, as discussed elsewhere (Tang and West, 2001). This should apply, even if the cause of the lag was restricted to the preimplantation stage, as long as the time lag persists after implantation. During gastrulation, the average cell cycle time is about 5-6 h, ranging from 3-3.5 h in the primitive streak to 7-7.5 h (Snow, 1976, 1977; Mac Auley et al., 1993). If BALB/c embryos continue to lag behind their partner embryos, in an aggregation chimaera, by 6 to 9 h during the early postimplantation period, BALB/c epiblast cells will lag at least a whole cell cycle behind and so would contribute significantly less to the chimaera.

Strain-specific selection pressures in chimaeras can affect the quantitative contributions to the whole chimaera, as described in the present study, or contributions to specific solid tissues (Mintz, 1970; Peterson, 1979) or blood (Mintz and Palm, 1969; Tuffrey et al., 1973; West, 1977). One report of C3H↔C57BL/6 chimeras indicated that C57BL/6 cells tended to predominate in certain tissues while C3H cells tended to predominate in others (Mintz, 1970), suggesting the presence of tissue-specific selection pressures. Chimaera experiments have also shown that some abnormal genotypes may be at a general selective disadvantage (e.g. *Gpi1^−/−^* null cells; Keighren et al., 2016) and others may be depleted in specific tissues (e.g. *Pax6^−/−^* null cells; Quinn et al., 1996). Lineage-specific selection occurs in tetraploid↔diploid chimaeras when tetraploid cells are excluded from the epiblast but not from the PrE or TE lineages (James et al., 1995; Eakin et al., 2005; MacKay and West, 2005).

Tetraploid or *Bmpr1a^−/−^* null embryonic stem (ES) cells are also eliminated from mixed cultures of ES cells by a competitive process, which occurs when pluripotent ES cells differentiate. This depends on the presence of wild-type cells and involves establishing lower levels of c-Myc in cells that are later eliminated by apoptosis (Sancho et al., 2013). Further work is required to determine whether this type of competitive interaction is involved in selecting against other cell types in chimaeras. Although it is possible that reduced cell numbers, produced by delayed or slow development of BALB/c embryos, is sufficient to explain their poor contribution to chimaeras, as discussed above, it is also possible that the type of competitive interaction described by Sancho et al. (2013) identifies cells from such embryos and marks some for elimination between E4.5 and E8.5.

Our conclusion, that the poor contribution of BALB/c cells to aggregation chimaeras is attributable to selection that is probably linked to the delayed or slow development of BALB/c embryos, may also be relevant to optimising germline transmission of ES cell genotypes via ES cell chimaeras. This is an important technical challenge for the efficient production of genetically modified mice. In one study, ES cell chimaeras with a large ES cell contribution were produced more efficiently if ES cells were combined with BALB/c blastocysts, from unsuperovulated 12-week-old females, rather than C57BL/6-albino or BALB/c blastocysts from females superovulated at 4 weeks (Esmail et al., 2016). Although this did not distinguish the effects of genotype, maternal age and induced superovulation, another study showed that BALB/c blastocysts were superior to C57BL/6-albino blastocysts for chimaera production with C57BL/6N-derived ES cells, when all the embryos were produced by females superovulated at 3-4 weeks (Alcantar et al., 2016). Although C57BL/6-albino females produced more blastocysts, use of BALB/c host blastocysts produced a higher birth rate, a higher yield of chimaeras, more male chimaeras with a high ES cell contribution and a higher rate of germline transmission of the ES cell genotype.

Alcantar et al. (2016) suggested that BALB/c strain embryos might provide a less competitive host environment than C57BL/6-albino embryos and so allow the C57BL/6N ES cells to contribute better to ES cell chimaeras. If BALB/c host blastocysts lagged behind C57BL/6-albino host blastocysts, this might allow more extensive colonisation by ES cells, even if BALB/c cells did not continue to divide more slowly. As XY, ES-derived cells are expected to colonise the germ line of male chimaeras if they contribute at least 30% overall (Fielder et al., 2012), selection that increased the contribution of ES cells would facilitate germline transmission. To further optimise the production of genetically modified mice via ES cell chimaeras, it may be worth evaluating other strains that develop slowly. If slow cell cycle times in preimplantation embryos can contribute to a selective disadvantage, candidate strains would include the ‘superslow’ BALB.K strain. This strain combines the uncharacterised effects of the BALB/c genetic background and the *Ped-slow* allele, so that preimplantation BALB.K embryos develop more slowly than BALB/c embryos (Goldbard and Warner, 1982).

## MATERIALS AND METHODS

### Mice

All animal work was performed in accordance with institutional guidelines and UK Home Office regulations (licences PPL 60/1150, PPL 60/1989 and 60/2887). The strains of mice (*Mus musculus* L.) used are summarised in Table 1. A/J/Ola/Hsd, BALB/c/Ola/Hsd and C57BL/OlaHsd strain mice were purchased from Harlan Olac Ltd, Bicester, UK. BALB/c/Eumm, C57BL/OlaWs (a separately maintained colony of C57BL/OlaHsd), CBA/Ca, (C57BL×CBA/Ca)F1 hybrids (abbreviated to ‘BF1’)_,_ the partially congenic C57BL-albino strain C57BL/Ola.AKR-*Gpi1^a^,Tyr^c^*/Ws (abbreviated to ‘BC’), (BC×BALB/c)F1 hybrids (abbreviated to AF1), (BALB/c×A/J)F1 hybrids (abbreviated to ‘AAF1’) and stocks ‘TGB’ and ‘TP6.3’ were bred and maintained under conventional conditions at the University of Edinburgh with a light cycle of 14 h light (05:00 h-19:00 h) and 10 h dark or 12 h light (06:00 h-18:00 h) and 12 h dark.

Stock TGB was homozygous for the reiterated *TgN(Hbb-b1)83Clo* transgene (abbreviated to *Tg*) (Lo, 1983; Lo et al., 1987) and was maintained as a closed stock on a predominantly CBA/Ca and C57BL/OlaWs genetic background as described elsewhere (Keighren et al., 2015). Stock TP6.3 was hemizygous for the *TgTP6.3* tauGFP transgene (Pratt et al., 2000; MacKay et al., 2005). Hemizygous *TgTP6.3^+/−^* transgenic mice were distinguished from non-transgenic mice by fluorescence microscopy of ear biopsies, obtained for mouse husbandry purposes and the stock was maintained by crossing to BF1 hybrid mice. Both TGB and TP6.3 stocks were also pigmented and homozygous *Gpi1^b/b^*. Mice used as embryo recipients for chimaera production were F1 hybrids (designated ‘CF1’ hybrids; Table 1) and were homozygous for albino (*Tyr^c/c^*) and *Gpi1^c/c^* as described previously (West and Flockhart, 1994).

### Superovulation and embryo collection

Female mice, 5-7 weeks old, were induced to ovulate by intraperitoneal injections of 5 i.u. PMSG (pregnant mare's serum gonadotrophin; Folligon, Intervet, Cambridge, UK) at 12:00 h, followed by 5 i.u. hCG (human chorionic gonadotrophin; Chorulon, Intervet), 48 h later. For collection of unfertilised oocytes, superovulated females were culled at approximately 10:00 h on the morning after the hCG injection, oocytes were collected from the oviduct and cumulus cells were dispersed in a solution of 100 units hyaluronidase (Sigma-Aldrich, Gillingham, UK) per ml of phosphate-buffered saline (PBS). For collection of embryos, females were housed individually with males overnight, and the following morning (designated E0.5) the female was checked to determine if a vaginal plug, indicative of mating, was present. Plugged females were killed by cervical dislocation on the appropriate day, depending on the age of embryos required, and preimplantation embryos were flushed from the reproductive tract, washed and kept in M2 (Quinn et al., 1982) or KSOM-H (Summers et al., 1995) handling medium until they were used.

### Production of chimaeric embryos

Mouse chimaeras were produced by aggregating pairs of genetically distinct, E2.5 day preimplantation embryos (Tarkowski, 1961; Mintz, 1962; Mintz et al., 1973), as previously described (West and Flockhart, 1994). The strain combinations were the same as those used for published E12.5 chimaera series 3U, 3B, 4U and 4B, as shown in Table 2 (Tang and West, 2001; MacKay et al., 2005). Some E3.5 chimaeric embryos in series 3U and 3B were transferred to E2.5 *Gpi1^c/c^*, CF1 hybrid strain, pseudopregnant recipients as described elsewhere (West and Flockhart, 1994; Tang and West, 2001). Chimaeras that were analysed at E6.5-E8.5 were removed from their decidual swellings with watchmaker forceps under a dissecting microscope. Embryos for analysis by GPI electrophoresis were stored at −20°C as described below.

### Analysis of chimaeric blastocysts with GFP marker

For the experiments with GFP blastocyst chimaeras, embryos were aggregated, cultured in KSOM medium (Summers et al., 1995) under mineral oil in an incubator (37°C, 5% CO_2_ in air) for 24 h, then transferred to fresh pre-equilibrated drops of culture medium under mineral oil in a WillCo HBSt-3522, thin glass-bottomed dish (Intracel Ltd., Royston, Herts, UK). This was placed in an environmental chamber on top of the pre-heated stage (THD 60, Linkam Scientific Instruments Ltd., Tadworth, UK) of a Leica DMIRB/E inverted confocal microscope, and the atmosphere within the chamber was maintained at 37°C, 5% CO_2_ in air, as described elsewhere (Sharp et al., 2017). Time-lapse images were acquired using the Leica TCS NT confocal system, and images from fluorescein isothiocyanate (FITC) and transmitted light channels were merged (Sharp et al., 2017). The percentage contributions of tauGFP-positive cells to the inner cell mass (ICM), polar trophectoderm (pTE; overlying the ICM) and mural trophectoderm (mTE; surrounding the blastocyst cavity) were estimated in chimaeric blastocysts. Regions of GFP fluorescence and non-fluorescence were measured in single optical sections from time-lapse images of early, mid and expanded blastocyst stages, as described elsewhere (MacKay and West, 2005).

### Analysis of E4.5 to E8.5 chimaeras by GPI electrophoresis

Oocytes and preimplantation embryos for analysis by GPI electrophoresis were collected individually or in groups of 3 or 6 in a small volume of M2 handling medium, held between two small volumes of paraffin oil, in finely drawn Pasteur pipettes and stored at −20°C. E6.5-E8.5 postimplantation embryos for analysis were stored in 50% glycerol in water in 96-well plates. Before electrophoresis, samples were lysed by three cycles of freezing and thawing and postimplantation samples were disrupted mechanically. The overall proportions of the *Gpi1^a/a^* and *Gpi1^b/b^* cell populations in chimaeric embryos were estimated from the proportions of GPI1A and GPI1B allozymes, by cellulose acetate electrophoresis, staining for GPI1 activity and densitometry with a Helena Process-24 gel scanner as previously described (West and Flockhart, 1994). Small samples were applied to the cellulose acetate plates from finely drawn Pasteur pipettes and larger samples were applied with the applicator. Any maternal cells from the CF1 strain recipient females were homozygous *Gpi1^c/c^*, so only produced a GPI1C band, which was excluded from the calculations.

### Embryo development experiments

Females were induced to ovulate (see above) and paired overnight with males. Preimplantation embryos were flushed from the reproductive tract of pregnant females at E2.5 and cultured singly in drops of M16 culture medium (Whittingham, 1971) under paraffin oil in bacteriological grade Petri dishes (Sterilin Ltd., Newport, UK) for a further 24 h. For the purpose of comparing the development of embryos from different genetic crosses, healthy embryos were classified according to the following numerical scale: (score 1) 2-cells, (2) 3-4 cells, (3) 5-8 cells, (3.5) compacting 5-8 cells or uncompacted morula (>8 cells), (4) compacted morula, (5) early blastocyst (cavity <50% total), (5.5) mid-blastocyst (cavity ∼50% total), (6) expanded blastocyst (cavity >50% total). The mean developmental score was calculated for each time point and the percentage of blastocysts was calculated at E3.5, at the end of the culture period. A relative developmental index for the *Gpi1^a/a^* strain in each chimaera combination was then calculated to compare the developmental scores or percentage blastocysts of the strains that were combined in E12.5 *Gpi1^a/a^*↔*Gpi1^b/b^* chimaeras. For example, if the mean E2.5 developmental scores for *Gpi1^a/a^* and *Gpi1^b/b^* embryos were respectively A and B, the relative developmental index for the *Gpi1^a/a^* versus *Gpi1^b/b^* strain comparison (equivalent to *Gpi1^a/a^*↔*Gpi1^b/b^* chimaeras) would be calculated as 100A/(A+B) and expressed as a percentage. In some experiments, cells of E2.5 embryos were counted directly with a dissecting microscope.

Three embryo development experiments were undertaken to compare the preimplantation development of the embryos with genotypes used in E12.5 chimaera experiments 1-3, shown in Table 2. Each embryo development experiment was repeated three times and the data were pooled. Embryo development experiments 1 and 2 were completed more than a year before experiment 3 and the timing of embryo collections differed by about 2 h. Mice used to provide embryos for development experiments 1 and 2 were housed in 14 h light (05:00 h-19:00 h) and 10 h dark and E2.5 embryos were collected at 10:30-11:45 h (at approximately 59 h after the middle of the dark period or 71 h after the hCG injection) and cultured for 24 h. Mice used to provide embryos for development experiment 3 were housed in 12 h light (06:00 h-18:00 h) and 12 h dark and E2.5 embryos were collected at 8:30-9:30 h (at approximately 57 h after the middle of the dark period or 69 h after the hCG injection) and cultured for 24 h.

### Statistics

Minimum group sizes were guided by previous experience and power calculations. For the compositions of E6.5-E8.5 chimaeras, chimaera group sizes were chosen to ensure sufficient power to detect, as significant (*P*<0.05), mean percentage GPI1A differences that were smaller than those previously published for E12.5 chimaeras of the same strain combination (shown as experiment 3 in Fig. S1 and Table 2). The choice of parametric or non-parametric tests was guided, in part, by normality tests. GraphPad Prism version 5.0c (GraphPad Software Inc., La Jolla, CA, USA) was used for most statistical tests. Results for two groups were analysed by Mann–Whitney *U*-tests. More than two groups were analysed by Kruskal–Wallis tests followed by Dunn's post tests or by Friedman tests followed by Dunn's post tests. The Spearman correlation coefficient was used to analyse relationships between two variables (embryo development and chimaera composition). An online statistical calculator (http://vassarstats.net/index.html) was used for Fisher's exact tests. Where data are plotted as box and whisker plots, they show the median (horizontal line within the box), upper and lower quartiles (top and bottom of boxes) and the minimum and maximum of all the data (ends of whiskers).

## Supplementary Material

Supplementary information
