# Supplementary material for: Selection against BALB/c strain cells in mouse chimaeras
Source: Biol Open. 2018 Jan 15;7(1):bio030189. doi: 10.1242/bio.030189 (PMC5829504; doi:10.1242/bio.030189)
Supplement: Supplementary information [file biolopen-7-030189-s1.pdf]

Supplementary Figure S1

Tang P-C, MacKay GE, Flockhart JH, Keighren MA, Kopakaki A and West JD

Selection against BALB/c strain cells in mouse chimaeras

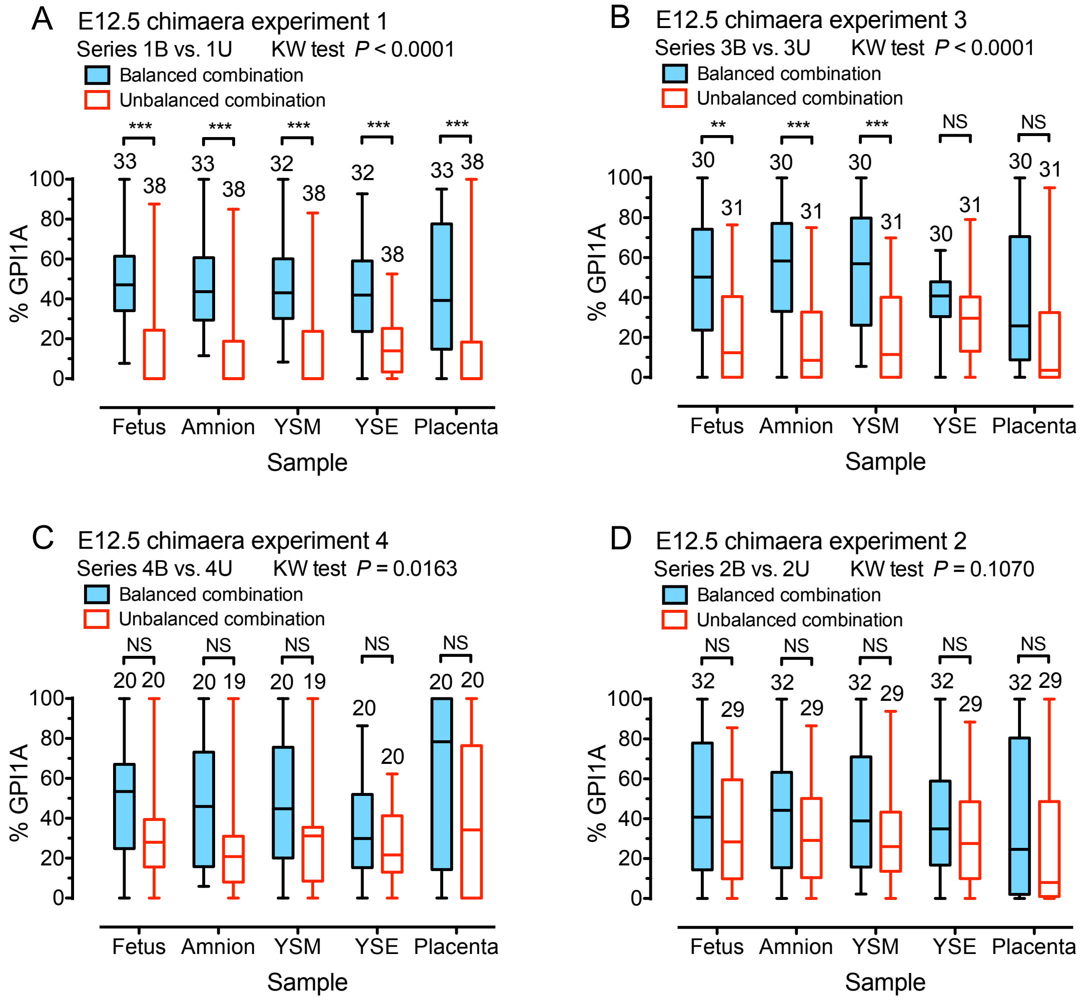

Figure S1. Percentage GPI1A contribution to E12.5 balanced and unbalanced series of chimaeras.

Results from four published studies, each comparing a series of balanced (B) and a series of unbalanced (U) E12.5 albino (*Tyr*$^{c/c}$), *Gpi1*$^{a/a}$↔pigmented (*Tyr*$^{+/+}$), *Gpi1*$^{b/b}$ chimaeras. The % GPI1A contribution to the fetus, amnion, yolk sac mesoderm (YSM), yolk sac endoderm (YSE) and placenta was estimated by glucose phosphate isomerase (GPI) electrophoresis. **(A-C)** Experiments 1, 3 and 4 each compared a series of BALB/c↔*Gpi1*$^{b/b}$ chimaeras with a series of F2 hybrid↔*Gpi1*$^{b/b}$ chimaeras (experiment 1) or F1 × transgenic strain ↔*Gpi1*$^{b/b}$ chimaeras (experiments 2 and 3), where the F1 hybrid mice were produced from crosses between BALB/c and another albino, *Gpi1*$^{a/a}$ strain. The strain combinations, mean % GPI1A values and references are shown in Tables 1 and 2. In each case BALB/c cells contributed less to the fetus, amnion, yolk sac mesoderm, yolk sac endoderm and placenta (series 1U, 3U and 4U) than the corresponding hybrid embryos (series 1B, 3B and 4B) and in many cases these differences were significant. **(D)** In experiment 2, the contribution of embryos produced by the reciprocal backcrosses between BALB/c and ‘AF1’ hybrid mice were compared. Backcross embryos with BALB/c mothers (series 2U) tended to contribute more poorly to chimaeras than reciprocal backcross embryos (series 2B) but this trend was not significant. Results for the Kruskal-Wallis (KW) test and Dunn’s multiple comparison test (asterisks) are shown. ** $P < 0.01$; *** $P < 0.001$; **** $P < 0.0001$; NS, not significant. The number of chimaeras is shown above the box and whisker plots.

Biology Open • Supplementary information

Table S1. Frequencies of non-chimaeric conceptuses in E6.5-E8.5 balanced series 3B and unbalanced series 3U

Age	Total conceptuses	Number of chimaeras	Number of non-chimaeras			Significance of difference (series 3B vs. 3U)	
			Total	0% GPI1A	100% GPI1A	Non-chimaera frequency*	0% GPI1A frequency†
**Balanced series 3B**							
E6.5	46	41	5 (11%)	3 (60%)	2	-	-
E7.5	53	45	8 (15%)	4 (50%)	4	-	-
E8.5	50	44	6 (12%)	3 (50%)	3	-	-
Total	149	130	19 (13%)	10 (53%)	9	-	-
**Unbalanced series 3U**							
E6.5	38	32	6 (16%)	5 (83%)	1	$P = 0.5342$ (NS)	$P = 0.5455$ (NS)
E7.5	42	27	15 (36%)	12 (80%)	3	$P = 0.0293$	$P = 0.1819$ (NS)
E8.5	39	32	7 (18%)	6 (86%)	1	$P = 0.5484$ (NS)	$P = 0.2657$ (NS)
Total	119	91	28 (24%)	23 (82%)	5	$P = 0.0241$	$P = 0.0503$ (NS)

* The chimaera : non-chimaera frequency ratio was compared between series 3B and 3U for all conceptuses by Fisher's exact test.

† The 0% GPI1A : 100% GPI1A frequency ratio was compared between series 3B and 3U for non-chimaeras by Fisher's exact test.

Table S2. Comparison of developmental stages at the time when E2.5 embryos were collected.

Series*	Strain (female x male)	Total embryos	Number of E2.5 Embryos 2-cell (score 1)†	3-4 cell (2)	5-8 cell (3)	>5-8 cell (3.5)	c-morula (4)	e-blast (5)	m-blast (5.5)	l-blast (6)	Mean E2.5 developmental score
**Embryo development experiment 1**											
1U	BALB/c	55	0	4	44	7	0	0	0	0	2.99
1B	AF2	72	0	0	26	46	0	0	0	0	3.32
1B & 1U	BF2	80	0	0	6	73	1	0	0	0	3.47
**Embryo development experiment 2**											
2U	BALB/c × AF1	35	0	2	28	5	0	0	0	0	3.01
2B	AF1 × BALB/c	48	0	0	19	29	0	0	0	0	3.30
2B & 2U	BF2	41	0	0	8	33	0	0	0	0	3.40
**Embryo development experiment 3‡**											
3U	BALB/c	66	0	23	43	0	0	0	0	0	2.65
3B	AAF2	75	0	1	45	29	0	0	0	0	3.18
3B & 3U	BF1 × TGB	83	0	0	51	32	0	0	0	0	3.19

* Series refers to the chimaera series shown in Table 2 that was made using embryos of the same strain

† Embryos were scored according to the following numerical scale: (1) 2-cells, (2) 3-4 cells, (3) 5-8 cells, (3.5) >5-8 cells (compacting 5-8 cells or uncompacted morula with >8 cells), (4) compacted morula (c-morula), (5) early blastocyst (e-blast; cavity < 50% total), (5.5) mid-blastocyst (m-blast; cavity ~50% total), (6) late or expanded blastocyst (l-blast; cavity >50% total). The mean developmental score was calculated for each time point.

‡ In embryo development experiments 1 & 2, embryos were collected at approximately 59 h. after the middle of the dark period (~71 h. after the hCG injection). In embryo development experiment 3, embryos were collected approximately 57 h. after the middle of the dark period (~69 h. after hCG). See Materials and Methods.

Table S3. Comparison of developmental stages for embryos that were collected at E2.5 and cultured for 24 hours to E3.5.

Series*	Strain (female x male)	Total embryos	Number of E3.5 Embryos								Mean E3.5 development score	Percentage blastocysts
			2-cell (score 1)†	3-4 cell (2)	5-8 cell (3)	>5-8 cell (3.5)	c-morula (4)	e-blast (5)	m-blast (5.5)	l-blast (6)		
**Embryo development experiment 1**												
1U	BALB/c	55	0	0	0	2	36	10	1	6	4.41	30.9
1B	AF2	72	0	0	0	0	8	23	12	29	5.38	88.9
1B & 1U	BF2	80	0	0	0	0	28	29	6	17	4.90	65.0
**Embryo development experiment 2**												
2U	BALB/c × AF1	35	0	0	0	2	17	9	0	7	4.63	45.7
2B	AF1 × BALB/c	48	0	0	0	1	3	3	0	41	5.76	91.7
2B & 2U	BF2	41	0	0	0	0	2	15	4	20	5.49	95.1
**Embryo development experiment 3‡**												
3U	BALB/c	66	0	3	5	4	29	20	5	0	4.22	37.9
3B	AAF2	75	0	0	0	0	12	29	22	12	5.15	84.0
3B & 3U	BF1 × TGB	83	0	0	0	0	53	29	1	0	4.37	36.1

* Series refers to the chimaera series shown in Table 2 that was made using embryos of the same strain

† Embryos were scored according to the following numerical scale: (1) 2-cells, (2) 3-4 cells, (3) 5-8 cells, (3.5) >5-8 cells (compacting 5-8 cells or uncompacted morula with >8 cells), (4) compacted morula (c-morula), (5) early blastocyst (e-blast; cavity < 50% total), (5.5) mid-blastocyst (m-blast; cavity ~50% total), (6) late or expanded blastocyst (l-blast; cavity >50% total). The mean developmental score was calculated for each time point.

‡ In embryo development experiments 1 & 2, embryos were collected at approximately 59 h. after the middle of the dark period (~71 h. after the hCG injection). In embryo development experiment 3, embryos were collected approximately 57 h. after the middle of the dark period (~69 h. after hCG). See Materials and Methods.

Biology Open • Supplementary information
